# Beverage Consumption in Reproductive-Age and Postmenopausal Mexican Women: Habits and Associated Factors

**DOI:** 10.3390/foods14173124

**Published:** 2025-09-06

**Authors:** Alexandra Tijerina, Daessy Newton-Rubi, Silvia García, Rogelio Salas, Cristina Bouzas, Josep A. Tur

**Affiliations:** 1 Center of Research in Nutrition and Public Health, School of Public Health and Nutrition, Universidad Autonóma de Nuevo León, Monterrey, Nuevo León 64460, Mexico; 2 Research Group on Community Nutrition and Oxidative Stress, University of Balearic Islands–IUNICS, 07122 Palma de Mallorca, Spain; 3 Health Institute of the Balearic Islands (IDISBA), 07120 Palma de Mallorca, Spain; 4 CIBER Physiopathology of Obesity and Nutrition (CIBEROBN), Institute of Health Carlos III (ISCIII), 28029 Madrid, Spain

**Keywords:** beverages, beverage intake, postmenopause, women, Mexico

## Abstract

Water consumption in adults usually reaches lower levels than the recommendations, and evidence of the beverage consumption habits of women in the stages around menopause is scarce. The aim of this study was to assess the consumption of beverages and to determine how physical, psychological, and environmental factors modify the hydration habits of reproductive-age and postmenopausal women in the northeast of Mexico. We carried out a cross-sectional study of 40–65-year-old female (n = 690) residents in the metropolitan area of Monterrey, Nuevo León state, Mexico, who were classified as reproductive (n = 263) and postmenopausal (n = 427). Anthropometrics, including body composition, beverage consumption, physical activity, and physical, psychological, and environmental factors, were assessed. There were no differences between the BMI and waist-to-height ratio of reproductive-age and postmenopausal women. The total daily beverage consumption did not differ between reproductive-age and postmenopausal women, with an average beverage consumption of 2723–2915 g/day. A third of the women studied consumed less than 1.5 L/day, and another third—mainly the younger participants—consumed between 1.5 and 2.0 L/day. The most consumed beverage was plain water, followed by regular soda, flavored beverages, coffee, and diet soda. Consumption of regular soda, flavored beverages, and milk was higher among reproductive-age women. The postmenopausal women indicated a higher consumption of plain water and juices. Similar effects of physical and psychological factors and environmental temperature on the beverage consumption of reproductive and postmenopausal women were observed. Physical activity, maximum daily temperature, and body composition were the factors that conditioned beverage intake.

## 1. Introduction

Physiological aging leads to changes in internal–external water levels and control mechanisms at both central and peripheral levels. Adequate hydration is achieved when the total fluid intake maintains a normal body water reservoir and distribution, capable of meeting the physiological demands of the body. Hydration requirements can be affected by several factors, such as environmental and body temperature, physical activity, dietary habits, and renal function [1]. The total water intake (TWI) can be obtained from three main sources: 70–80% from beverage consumption (water, coffee, tea, juices, soft drinks, etc.), 20–30% from food intake, and 10% from endogenous oxidation of macronutrients [2].

The recommendations for daily water intake consumption are different in different countries. The Mexican Ministry of Health recommends drinking 2 L of water daily [3] based on the proposed Jarra del Buen Beber (Well-drink Jar), suggesting that the general population drink six to eight glasses of water daily, while other beverages, such as sweet and alcoholic beverages and sodas, are not recommended [4]. The European Food Safety Authority proposes that women should drink 2 L of water daily [5], while the U.S. Institute of Medicine recommends that 19–70-year-old women drink 2.7 L of water per day [6]. Others have suggested an individualized approach, with the appropriate amount of fluid intake set at 33 mL/kg/day, evenly distributed over the day [7]. Individual requirements could vary widely according to personal characteristics, such as age, size, body composition, physical activity, and environmental factors [8].

The Mexican National Health and Nutrition Survey (ENSANUT) reported an average national consumption of plain water in only 85.9% of the adult population and 87.6% of women, while the other highly consumed beverages were sweetened non-dairy beverages (83.6%) [9]. Water consumption in adults is much lower than the recommendations, only reaching 2.5 cups daily (600 mL/d) [10], which could contribute to the compensatory consumption of other beverages, like sweetened ones. A total of 66.8% of Mexican adult women reported consuming sweetened beverages between the years 2020 and 2022 [11].

There is scarce evidence for the beverage consumption habits of women in the stages around menopause. Therefore, the aim of this study was to assess the consumption of beverages in reproductive-age and postmenopausal women in the Northeast of Mexico and to determine how physical, psychological, and environmental factors modify hydration habits.

## 2. Methods

### 2.1. Design and Study Participants

**Figure 1:**
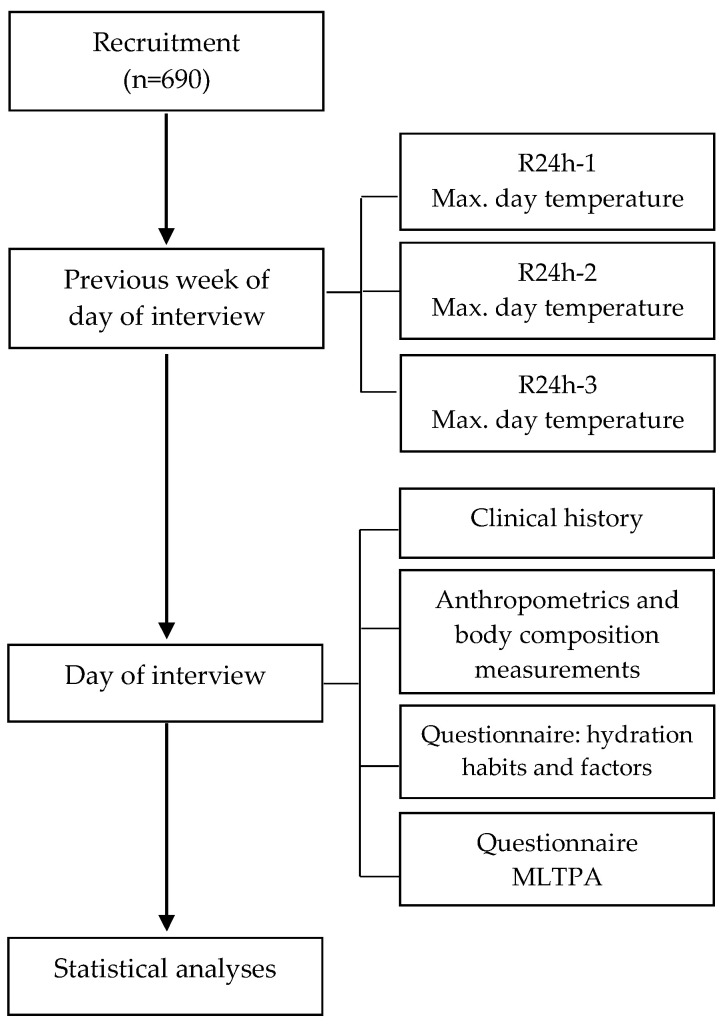
Flowchart of study protocol. MLTPA: Minnesota Leisure Time Physical Activity.

A descriptive cross-sectional study was carried out during 2015–2019. The sample size for this study was calculated from a total of 814,632 women, aged 40–65 years old, who were residents of the metropolitan area of Monterrey, Nuevo León state, Mexico [12]. To demonstrate the consumption of beverages and sociodemographic characteristics with a statistical power of 80%, and accepting an alpha risk of 0.05 and a beta risk of 0.2 on a two-sided test, 100 subjects were necessary in each (reproductive-age and postmenopausal) group to recognize a minimum difference of 1 unit between the groups as significant. The common deviation was assumed to be 1.4, and a drop-out rate of 10% was anticipated. Women were invited to participate via social media, via flyers posted at medical centers, universities, and public areas, and via direct invitation outside supermarkets and department stores. Voluntary response sampling was followed for the recruitment. The exclusion criteria were any disease related to avoiding natural feeding, pregnancy, abandonment of the study, and missing or incomplete data. A final sample of 690 women participated in this study (Figure 1) and were considered to be representative of the population. Software used: http://www.imim.es/ofertadeserveis/software-public/granmo/Version 7 (accessed on 12 April 2012).

This study followed the Declaration of Helsinki and was approved by the Ethics Committee of the Facultad de Salud Pública y Nutrición (Protocol ID: 15-FaSPyN-SA-11). Written informed consent was obtained from participants. Interviews and assessments were performed at the Center for Research in Nutrition and Public Health of the Facultad de Salud Pública y Nutrición.

### 2.2. Clinical History

Clinical history of participants included date of birth and age, use of drugs, date of last menses, and duration of, and changes in, menstrual cycles according to STRAW+10 criteria [13]. Women were classified into two stages: (1) reproductive-age—regular cycles or non-noticeable changes were presented (n = 263); (2) postmenopausal—the absence of a menstrual cycle for the last ≥12 months (n = 427).

### 2.3. Anthropometrics and Body Composition

Height was measured using a digital stadiometer (SECA 274, ±2.0 mm, SECA, Mexico DF, Mexico), with the participant’s head in the Frankfurt plane. Weight was measured using a scale (SECA 874, ± 0.1 kg, SECA, Mexico DF, Mexico). Body mass index (BMI) was determined by the formula BMI = weight (kg)/height^2^ (m^2^), and subject were classified as obese when ≥30 kg/m^2^, overweight when 25–29.9 kg/m^2^, or normal weight when 18.5–24.9 kg/m^2^ [14,15]. Waist circumference (WC) was measured using a non-stretch measuring tape (SECA 201, ±0.1 cm, SECA, Mexico DF, Mexico) at the midpoint between the last rib and the iliac crest. Waist-to-height ratio (WHtR) was determined by the formula WHtR = waist circumference (cm)/height (cm).

Total body fat and free-fat mass percentages (%) were measured using bioelectrical impedance analysis, Inbody A120, and Software Lookin’Body v.120 (Microcaya, Bilbao, Spain; https://www.composicion-corporal-inbody.com/LookinBody.html, accessed on 11 June 2025), based on eight electrodes and two frequencies (20 kHz and 100 kHz).

### 2.4. Beverage Consumption

Three-day food and beverage recalls (R24h-1, R24h-2, and R24h-3) were used—two weekdays and one weekend day—for the week before participants’ interviews and measurements. Women were asked to report detailed food and beverage consumption information for the previous day of recall, including information on preparation, brands, and the amounts consumed. The maximum day temperature (°C) of the day of recall was also reported.

According to the Guide for a Healthy Hydration of the Spanish Society of Community Nutrition [16], the beverages were categorized into plain water, flavored beverages, fruit juices, diet soda, milk, yogurt, coffee, tea, regular soda, energy drinks, beer, wine, and alcoholic distilled beverages (Supplementary Table S1).

Total energy intake (TEI) (kcal/d), amount of beverage consumption (g/d), total water intake (TWI) from foods and beverages (g/d), and consumption of alcohol (g/d) and caffeine (mg/d) were analyzed in the software Food Processor^®^ version 15.0 (ESHA Research, Salem, OR, USA). Contribution of beverages and foods to TWI (g/d) and TEI (kcal/d) was also calculated as a percentage.

### 2.5. Hydration Factors and Habits

A questionnaire was answered to evaluate general beverage consumption habits and physical, psychological, and environmental factors affecting consumption. It included a question to determine participants’ general habits—in which they could select only one of four possible answers: (1) “I drink below 1.5 L daily (L) and try to consume more”; (2) “I try 1.5 to 2.0 L per day but cannot achieve it”; (3) “I drink from 1.5 to 2.0 L daily”; and (4) “I drink above 2.0 L per day”—and a last question to determine changes in beverage consumption in the past 5 years (Yes/No/Don’t know).

Physical factors, such as loss of thirst, taste, and smell, were reported by participants (Yes/No/Indifferent/Don’t know). They were considered as factors reflecting beverages consumption at the time of interview. A section on psychological factors was included to determine the use of reminders for increasing beverage consumption (Yes/No). The use of specific reminders was also reported (Yes/No), such as (1) a jar or glass of water or another beverage in a visible place (at daytime); (2) a note (on the fridge or another visible place); (3) a glass of water or another beverage on the bedside table (at night); and (4) carrying a bottle all the time.

An environmental factor, namely, the average maximum day temperature (°C) across the three days of food and beverages recall, was also calculated.

### 2.6. Minnesota Leisure Time Physical Activity Questionnaire

An estimated level of physical activity was determined from the reported activities listed in the Minnesota Leisure Time Physical Activity questionnaire (MLTP) [17,18]. The questionnaire was adapted to Mexican women and contains a total of 63 physical activities divided into 8 groups: walking/dancing/climbing, general maintenance activities, aquatic activities, winter activities, sports, gardening, domestic activities, and fishing/hunting. Each activity from the questionnaire had its own physical intensity metabolic equivalents of task (MET) code via The Compendium of Physical Activities [19]. During the interview, participants reported the frequency (days per week) and duration (minutes per day) of the activities performed during the previous week. Energy expenditure in physical activity (EEPA) was reported in total daily MET (MET/d).

### 2.7. Statistics

Statistical analyses were performed using IBM SPSS^®^ Statistics software (v.25, SPSS Inc., Chicago, IL, USA). Data were analyzed for normality by the Kolmogorov–Smirnov test. Descriptive statistics are shown for participants’ characteristics. Mean and standard error of the mean (SE) were determined as numerical variables, and the number of responses (n) and the percentages of those response (%) were calculated as categorical variables. Differences between groups in different stages of reproductive aging (i.e., reproductive-age vs. postmenopausal) were analyzed by an independent *t*-test (equal variances) for numerical data and by a Chi-squared test (*χ*^2^) for categorical data.

Multiple linear regression models were used to assess the association between total beverage consumption (g/d) (dependent variable) and total energy expenditure in physical activity (MET/d), maximum day temperature (°C), body fat percentage (%), and free-fat mass percentage (%) (independent variables). The association of one unit increase for each of these parameters with total beverage consumption (g/d), with SE and 95% confidence interval (95%CI), were also calculated. Model 1 was unadjusted; model 2 was adjusted for age (years), BMI (kg/m^2^), and total energy intake (kcal/d); and model 3 was adjusted for age (years), BMI (kg/m^2^), energy (kcal/d), caffeine (mg/d), alcohol (g/d) intakes, and use of medication (yes/no). The level of significance was set at *p* < 0.05.

## 3. Results

A total of 690 women participated in this study. Table 1 shows the characteristics of all participants grouped according to reproductive aging stage: reproductive-age (n = 263) and postmenopausal (n = 427) women. Reproductive-age women were, on average, 46.5 years old, and postmenopausal women group, on average, 53.8 years old (*p* < 0.001). BMI did not differ between reproductive stages. Waist-to-hip ratio (WHR) was higher in postmenopausal women (*p* = 0.008). Waist-to-height ratio (WHtR) did not differ between groups. Use of drugs was reported more frequently in postmenopausal vs. reproductive-age women (*p* < 0.001).

Table 2 shows the average total daily consumption of beverages and the contribution of individual beverages. Average beverage consumption was between 2723 and 2915 g per day in women, with no difference between stages (*p* = 0.055). The most consumed beverage was plain water (48.2 to 52.6% of total daily beverage consumption), followed by regular soda, flavored beverages, coffee, and diet soda, while the least consumed were wine and distilled beverages (≤1% of total daily consumption). Milk consumption was between 5.1 and 5.7% of beverage daily consumption.

Individual beverage consumption was higher in reproductive-age women, and significant for regular soda (*p* = 0.049), flavored beverages (*p* = 0.009), and milk (*p* = 0.040). Postmenopausal women showed non-significantly higher consumption of plain water (1432.8 vs. 1404.3 g/d) and juice (127.7 vs. 107.8 g/d).

Contribution of beverage and food consumption to total water intake (TWI) (g/d) and total energy intake (TEI) (kcal/d) according to reproductive stage is shown in Table 3. Water from beverages was 85.6% for reproductive-age and 80.1% for postmenopausal women, showing no differences between stages, whereas water from foods was lower in reproductive-age women (*p* < 0.01). Energy intake from beverages was higher in reproductive-age vs. postmenopausal women (271.2 vs. 239.0 kcal/d, respectively (*p* < 0.05)). In general, TWI and TEI did not differ between the two groups of women.

Table 4 shows the general beverage consumption habits and physical factors and psychological factors related to beverage consumption. There were no differences in responses between reproductive-age and postmenopausal women. In general, 26.7 to 31.2% of women reported consuming below 1.5 L per day and that they were trying to consume more beverages, while 28.1 to 33.5% consumed between 1.5 and 2.0 L of beverages per day. Most women (57.8 to 60.5%) had changed their consumption habits in the past 5 years. Physical factors that may influence consumption habits include loss of thirst, taste, and smell; however, only 11.0 to 11.8% of women reported a loss of thirst, while 5.3 to 10.1% reported a loss of taste and smell.

The use of reminders for increasing beverage consumption was a psychological factor reported by 45.2% of study participants. Some of these reminders included having a glass of water or another beverage in a visible place during the daytime, which was used by 22.4 to 24.1% of women, and carrying a bottle at all the times, which was reported by 16.7 to 17.8% of participants. The least frequently reported reminder was displaying a note (<2.5%).

Multiple linear regression models are shown in Table 5. Significant associations were found between total energy expenditure in physical activity (EEPA), body fat percentage, and total beverage consumption for reproductive-age women across all models. An increase of one unit of total EEPA (MET/d) was associated with an increase of 0.598 to 0.663 g/d of total beverage consumption (*p* < 0.001). Body fat percentage was significant in both reproductive-age and postmenopausal women across the three models, showing that an increase of 1% body fat may suggest an increment in total beverage consumption of 151.1 to 197.5 mL/d.

In postmenopausal women, the four analyzed independent variables were associated with total beverage consumption, as shown in all models. An increase in temperature of degree Celsius was associated with an increase of 35.903 to 38.584 g/day in terms of beverage consumption (*p* < 0.001), while an increase of one MET/d was associated with an increase of 0.486 to 0.570 g/day (*p* < 0.001). Free-fat mass was also associated with beverage intake (*p* < 0.001); and an increase in free-fat mass (%) corresponded to an increase of 174.0 to 182.1 g/day.

## 4. Discussion

This study assessed a cohort of 690 women stratified into reproductive-age and postmenopausal age groups, aiming to determine the associations between beverage consumption, general habits, and physical, psychological and nutritional factors. Body mass index (BMI) was comparable between groups, but this study failed to differentiate between lean and fat mass. These indicators reflected a higher percentage of body fat in postmenopausal women but not of fat-free mass (*p* = 0.058). Waist-to-hip-ratio (WHR), as an indicator of central adiposity and a sensitive marker of cardiometabolic risk [20], was higher in postmenopausal women. This was expected, as visceral adiposity increases during and after the menopausal transition. WHR >0.85 and waist-to-height ratio (WHtR) >0.5 [21,22], as well as body fat >40%, indicated an excess of body fat [23], reflecting cardiovascular risk for both reproductive-age and postmenopausal women. Pharmacological use was higher in postmenopausal women, suggesting increased metabolic abnormalities with aging.

The average total daily beverage consumption ranged from 2723 to 2915 g per day, with no significant differences between groups, although it was lower in postmenopausal women. This aligns with previous studies demonstrating that fluid intake decreases with menopause as aging women maintain thirst sensitivity to osmotic stimuli but lose some thirst sensitivity to changes in central body fluid volume due to variations in the hormonal levels of estrogen and progesterone [24,25]. The results showed that plain water accounted for 48.2% and 52.6% of total beverage consumption among reproductive-age and postmenopausal women, respectively. Considering the Mexican established guideline La Jarra del Buen Beber (The Well-drink Jar), plain water intake was, on average, below 6–8 cups (1440–1920 mL) daily, although total beverage consumption recommendation per day was met. A previous study in Spanish 46–65-year-old women reported a total beverage consumption of 1290.9 g/day [26], while Mexican female adults had a total fluid intake of 1748 mL/day [27]. Increasing plain water consumption up to 2 L/day has been suggested to bring about a notable decrease in systolic blood pressure, as demonstrated in a previous study of 50–75-year-old adults [28].

Reproductive-age women were also more inclined toward beverages with added sugar (regular soda) and flavor (flavored beverages), as well as also towards milk consumption, than postmenopausal women, showing higher total energy intake (TEI) from beverages. Lower TEI in the postmenopausal group may reflect healthier or lower-calorie beverage options, even when total fluid consumption (TWI) remains unchanged. These findings may help to enhance overall nutrition and weight management at different reproductive stages, thus maintaining long-term metabolic health through menopausal transition and the postmenopause stage.

The reliance on food sources for hydration as women age is also suggested and has been reported in other countries, such as Spain, France, and Italy, where TWI from foods ranged from 31.99 to 42.2% and from beverages from 55.8 to 68.61% [29]; however, the women’s ages were not as specific as in the current study, in which a TWI contribution of beverages from 80.1 to 85.6% was found. Previous studies reported a 18.3% contribution to TEI from beverages in 35–65-year-old African American women living in the United States [30], which is higher than our results (8.9 to 10.4%). Other beverage consumption did not attain significant differences between stages; however, it is well known that the consumed beverages should be evaluated during the menopausal transition and postmenopause, which may be relevant for bone health; i.e., the intake of milk and yogurt was associated with higher bone mineral density (BMD) of the femoral neck and lumbar spine among subjects with normal vitamin D (25(OH)D) concentration [31], indicating calcium and vitamin D as essential nutrients for bone health. On the contrary, a reduction in the consumption of carbonated beverages such as sodas may decrease the intake of sugars and phosphoric acid, which can be detrimental for bone mass [32].

Beverage consumption habits, as well as physical and psychological factors, resulted in no differences between the stages of reproductive aging. Between 26.7 and 31.2% of women reported consuming less than 1.5 L/day, and 28.1 to 33.5% reported a consumption between 1.5 and 2.0 L/day. These proportions indicate that a considerable population of women may not meet the hydration guidelines but are motivated to improve their intake. A relevant observation is that most participants, i.e., 57.8% to 60.5%, reported having modified their beverage consumption habits over the past five years. This trend may reflect an increase in the recognized importance of hydration in health and wellbeing.

Changes in consumption patterns over time were observed in a younger population (18–45-year-old), suggesting links to sociodemographic variables, such as sex, age, educational level, and place of living [33], and improvements in the risk indicators for the prevention of disease [28].

During menopause, the decrease in estrogen may alter taste perception (dysgeusia) and bring about dry mouth (xerostomia) and burning sensations, substantially impacting daily life, including chewing, swallowing, an altered preferences for sweetness [34,35], and palatal sensitivity, mainly in postmenopausal women [34]. In the current study, there were no differences between groups in terms of physical or psychological factors; therefore, these determinants did not appear to have an effect on beverage consumption in this cohort.

Less than 50% of women reported their use of reminders to increase beverage consumption, and they also indicated that other factors could be important determinants of their actual consumption behavior, such as environmental and physical activity. However, the modification of this behavior could help women undergoing menopausal transition to reach the recommended hydration target, as previous studies have reported that 5-week reminder interventions successfully increase water intake by an average of 29% [36]. Nutrition health educators and policymakers could also help women to reach their recommended hydration targets via education campaigns aimed at improving beverage consumption and general health habits.

Multiple regression models demonstrated that the studied variables are key factors in beverage consumption: physical activity (EEPA, MET/d), maximum day temperature (°C), and body composition indicators, i.e., fat and free-fat mass [23]. It is well known that individuals who undergo more physical exercise tend to report a higher intake of water [8,37] and other beverages such as fruit juices and sodas [38]. Study findings suggest that low increments in daily energy expenditure (1 MET/d) are linked to a proportional increase in beverage intake, reflecting physiological demands for thermoregulation and rehydration during physical activity [39].

Regression models reflect a slightly smaller effect on beverage consumption in postmenopausal compared to reproductive-age women, even though physical activity was reported to be slightly higher in postmenopausal women (510.3 vs. 490.2 MET/d) (*p* = 0.523). The difference in association coefficients between groups may be indicative of age-related physiological variations, mainly at the postmenopausal stage, in which women often experienced alterations in sweat gland function and vascular responses to thirst, which could lead to increased risk of dehydration due to a slower rate of body fluid replenishment [40]. Maximum environmental temperature (°C) also played a significant role in beverage consumption in postmenopausal women, as higher temperatures accentuate the need for body temperature regulation, which may have an effect in terms of the selection of cooler beverages, which also provide higher palatability [41]. In the current study, beverage consumption did not change during different seasons; this could be due to similar maximum environmental temperatures during winter (27.3 °C), spring (29.6 °C), and fall (28.6 °C) over the duration of the study, although summer, naturally, reached a higher maximum environmental temperature (34.8 °C). An effect on beverage consumption—i.e., 35.9 to 42.8 g/d—was demonstrated in postmenopausal women due to an increase of 1 °C in the maximum day temperature. A study of Japanese adults reported that water intake from beverages increased by only 8.4 g/d following an increase in outdoor temperature of 1 °C. The study in question recorded maximum day temperatures across the seasons ranging from 11.3 °C to 31.5 °C [42], which are lower than those of the region studied in this paper.

Changes in body composition during the menopausal transition and the postmenopausal stage are meaningful factors influencing beverage consumption. The current results demonstrated a positive association between body adiposity and total beverage consumption in both reproductive-age and postmenopausal women. Each 1% increase in body fat was associated with an additional 151.1 to 197.5 g/d intake of fluids across the multivariable models. The mechanisms that may explain these findings are those needed to increase fluid intake and to achieve osmotic balance in individuals with a higher body fat percentage. The percentage of fat-free mass was also an important factor, with a 1% increase being associated with an increment in beverage consumption of 174.0 to 182.1 g/d, although this finding was only significant for postmenopausal women. This finding was expected, as higher lean body mass increases basal metabolic rates and heat production; thus, water demand increases for body thermoregulation and as a substrate for metabolism [43,44]. Assessing body fat and lean mass may also be useful to develop individualized recommendations for women’s hydration and beverage consumption.

### Strengths and Limitations

The main strength of this study is that it provides relevant findings for beverage consumption in women during the reproductive-age and postmenopausal stages (40–65 years old). As for its limitations, the individual contribution of selected drinks to total water intake and total energy intake was not assessed in the main statistical analyses. Three 24 h food recalls were used to assess food and beverage consumption, as is usual in this kind of study; however, interviews are subject to random and systematic errors due to recall and reporting biases, as well as potential under-reporting and issues pertaining to poor recollection on the part of the participants. Finally, this study had a cross-sectional design that limits causal inference; a longitudinal study design is recommended for future studies.

## 5. Conclusions

Beverage consumption was assessed in 40–65-year-old Mexican women, and the results demonstrated that total daily beverage consumption was no different between reproductive-age and postmenopausal women. Despite the recommended beverage intake for women (2.0 L/day), a third of the participants consumed less than 1.5 L/day, and another third—mainly the younger participants—consumed between 1.5 and 2.0 L/day. The need to promote the following of the practical guidelines for plain water consumption is supported by the scientific evidence, demonstrating a decreased risk of adverse health conditions during menopause. General habits did not differ between reproductive-age and postmenopausal women; however, it is important to note that physical activity, maximum day temperature, and body composition are factors that condition beverage consumption. Further research may include the implementation of a longitudinal analysis, including a focus on specific types of beverages of particular relevance to this group, and which may relate to metabolic conditions such as bone health.

## Figures and Tables

**Table 1:** Characteristics of study participants grouped according to reproductive aging stage.

	Reproductive (n = 263)	Postmenopause (n = 427)	*p*
Age (years)	46.5 (0.2)	53.8 (0.2)	<0.001
BMI (kg/m^2^)	29.1 (0.5)	29.4 (0.3)	0.540
Waist-to-hip ratio (WHR)	0.94 (0.00)	0.95 (0.02)	0.008
Waist-to-height ratio (WHtR)	0.56 (0.004)	0.56 (0.004)	0.615
Body fat (%)	41.1 (0.4)	42.1 (0.3)	0.043
Free-fat mass (%)	58.9 (0.4)	58.0 (0.3)	0.058
Physical activity (MET/d)	490.2 (23.4)	510.3 (20.0)	0.523
	n (%)	n (%)	
Marital status			0.001
Married	211 (80.2)	286 (67.0)	
Divorced or separated	25 (9.5)	76 (17.8)	
Single/civil partnership	24 (9.1)	38 (8.9)	
Widowed	3 (1.1)	27 (6.3)	
Education level			0.002
Elementary	32 (12.1)	87 (20.4)	
High school or technician	102 (38.8)	147 (34.4)	
University	93 (35.4)	145 (34.0)	
Graduate	36 (13.7)	48 (11.2)	
Employment status			0.001
House care	108 (41.1)	142 (33.3)	
Employed	131 (49.8)	198 (46.4)	
Unemployed	21 (8.0)	34 (7.9)	
Retired	3 (1.1)	53 (12.4)	
Smoking			0.392
Yes	18 (6.8)	37 (8.7)	
No	245 (93.2)	390 (91.3)	
Use of medication			<0.001
Yes	107 (40.7)	239 (56.0)	
No	156 (59.3)	188 (44.0)	

Numeral data is shown as the mean (standard error of the mean), eith differences determined by independent *t*-test. Categorical data is shown as frequency (percentage), n(%), with differences determined by Chi-squared (*χ*^2^) test.

**Table 2:** Beverage consumption (g /day) by participants according to reproductive aging stage.

Type of Beverage	Reproductive (n = 263)		Postmenopause (n = 427)		*p*
	Mean (SE)	%	Mean (SE)	%	
Total	2915.3 (80.1)	100.00	2723.1 (61.2)	100.00	0.055
Plain water	1404.3 (60.7)	48.2	1432.8 (47.7)	52.6	0.712
Regular soda	270.2 (28.8)	9.3	205.0 (16.3)	7.5	0.049
Flavored beverages	265.9 (26.6)	9.1	186.7 (11.8)	6.9	0.009
Coffee	227.1 (16.9)	7.8	209.3 (11.3)	7.7	0.363
Diet soda	183.1 (23.9)	6.3	162.6 (16.1)	6.0	0.460
Milk	164.9 (11.0)	5.7	137.8 (7.8)	5.1	0.040
Fruit juices	107.8 (14.6)	3.7	127.7 (11.8)	4.7	0.294
Tea	99.6 (12.5)	3.4	96.6 (11.0)	3.6	0.859
Yogurt and milk beverages	91.7 (8.9)	3.1	84.4 (8.2)	3.1	0.559
Beer	57.2 (14.9)	2.0	45.6 (11.0)	1.7	0.525
Energy drinks	21.7 (6.2)	0.7	15.5 (4.9)	0.6	0.432
Wine	12.6 (3.5)	0.4	11.2 (3.1)	0.4	0.768
Alcoholic distilled beverages	9.1 (4.6)	0.3	7.9 (2.9)	0.3	0.812

Consumption, in grams per day (g/d), is shown as the mean (standard error of the mean) and percentage of total consumption per day (%).

**Table 3:** Contribution of beverages and foods to total water (g/d) and energy intake (kcal/d) by reproductive stage.

Stage	Water from Beverages		Water from Foods		Total Water Intake (TWI)	Energy from Beverages		Energy from Foods		Total Energy Intake (TEI)
	Mean (SE)	%	Mean (SE)	%	Mean (SE)	Mean (SE)	%	Mean (SE)	%	Mean (SE)
Reproductive age (n = 263)	2300.5 (69.7)	85.6	386.5 (88.5) **	14.4	2687.0 (78.9)	271.2 (16.7) *	10.4	2344.6 (49.0)	89.6	2615.8 (50.8)
Postmenopausal										
(n = 427)	2224.1 (54.3)	80.1	552.5 (67.5)	19.9	2776.6 (55.1)	239.0 (11.2)	8.9	2434.1 (38.8)	91.1	2673.1 (38.6)

Data is shown as the mean (standard error of the mean) and percentage (%). Differences between groups are shown as * *p* < 0.05; ** *p* < 0.01.

**Table 4:** Beverage habits and physical and psychological factors related to participants’ beverage consumption.

	Reproductive-Age (n = 263)	Postmenopausal (n = 427)	*p*
	Response (%)	Response (%)	
General consumption			
Consumption habits			0.194
Below 1.5 L and trying to consume more	82 (31.2)	114 (26.7)	
Try 1.5 to 2.0 L but cannot achieve	64 (24.3)	98 (23.0)	
Consume 1.5 to 2.0 L	74 (28.1)	143 (33.5)	
Consume above 2.0 L	43 (16.3)	72 (16.9)	
*p*	0.009	<0.001	
Change in consumption in past 5 years			0.460
Don’t know	5 (1.9)	6 (1.4)	
Yes	159 (60.5)	247 (57.8)	
No	99 (37.6)	174 (40.8)	
*p*	<0.001	<0.001	
Physical factors			
Loss of thirst			0.422
Don’t know	2 (0.8)	2 (0.5)	
Yes	31 (11.8)	47 (11.0)	
Indifferent	161 (61.2)	255 (59.7)	
No	69 (26.2)	123 (28.8)	
*p*	<0.001	<0.001	
Loss of taste and smell			0.629
Don’t know	1 (0.4)	0 (0.0)	
Yes	14 (5.3)	43 (10.1)	
Indifferent	182 (69.2)	263 (61.6)	
No	66 (25.1)	121 (28.3)	
*p*	<0.001	< 0.001	
Psychological factors			
Use of reminder for increasing consumption			0.990
Yes	119 (45.2)	193 (45.2)	
No	144 (54.8)	234 (54.8)	
*p*	<0.001	<0.001	
Reminders *			
Glass of water or other	59 (22.4)	103 (24.1)	0.612
beverage in a visible place (daytime)			
Reminder note	4 (1.5)	10 (2.3)	0.458
Glass of water on the bedside	17 (6.5)	20 (4.7)	0.314
table (at night)			
Carrying a bottle all the time	44 (16.7)	76 (17.8)	0.720
*p*	<0.001	<0.001	

Data is shown as the number of responses (in brackets, the percentages from the total sample of each group, i.e., reproductive-age or postmenopausal women, are shown). Differences are determined by Chi-squared (*Χ*^2^) test. * Participants could select either one or several types of reminder.

**Table 5:** Multiple linear regression models of association between total beverage consumption (g/d), total energy expenditure in physical activity (MET/d), and maximum day temperature (°C) in study participants according to reproductive aging stage.

	Reproductive		Postmenopause	
	(n = 263)		(n = 427)	
	B (SE) (95% CI)	*p*	B (SE) (95% CI)	*p*
MODEL 1				
Total EEPA (MET/d)	0.598 (0.208) (0.188, 1.008)	0.004	0.486 (0.144) (0.203, 0.769)	0.001
Maximum day temperature (°C)	26.747 (13.321) (−0.517, 52.976)	0.246	42.811 (10.540) (22.095, 63.528)	<0.001
Body fat (%)	182.9 (56.2) (72.5, 293.5)	0.001	197.5 (51.9) (95.1, 299.9)	<0.001
Free-fat mass (%)	−16.44 (23.9) (−63.8, 30.9)	0.493	182.1 (50.2) (83.1, 281.1)	<0.001
MODEL 2				
Total EEPA (MET/d)	0.602 (0.207) (0.194, 1.011)	0.010	0.570 (0.145) (0.285, 0.855)	<0.001
Maximum day temperature (°C)	21.573 (13.356) (−4.728, 47.875)	0.251	38.584 (10.504) (17.938, 59.230)	<0.001
Body fat (%)	151.1 (55.1) (42.7, 259.4)	0.006	153.6 (51.5) (52.1, 255.1)	0.003
Free-fat mass (%)	−27.4 (33.9) (−93.6, 38.9)	0.414	174.8 (49.1) (77.9, 271.6)	<0.001
MODEL 3				
Total EEPA (MET/d)	0.663 (0.206) (0.257, 1.069)	0.004	0.553 (0.143) (0.271, 0.834)	<0.001
Maximum day temperature (°C)	20.359 (13.268) (−5.770, 46.488)	0.229	35.903 (10.516) (15.232, 56.573)	0.001
Body fat (%)	151.8 (55.3) (43.0, 260.6)	0.006	154.7 (51.5) (53.3, 256.1)	0.003
Free-fat mass (%)	−28.1 (33.8) (−95.3, 39.0)	0.407	174.0 (49.1) (77.3, 270.7)	<0.001

Abbreviations: B: mean value; SE: standard error of the mean; CI: confidence interval; EEPA: total energy expenditure in physical activity; MET/d: metabolic equivalents of task per day. Model 1: unadjusted; Model 2: adjusted for age (years), BMI (kg/m^2^), and energy intake (kcal/d); Model 3: adjusted for age (years), BMI (kg/m^2^), energy intake (kcal/d), caffeine (g/d), alcohol (g/d), and use of medication (yes/no).

## Data Availability

There are restrictions on the availability of the data used for this trial due to the signed consent agreements around data sharing, which only allow access to external researchers for studies adhering to this project’s purposes. Individuals wishing to access the trial data used in this study can make a request to pep.tur@uib.es.

## Supplementary Materials

The following supporting information can be downloaded at https://www.mdpi.com/article/10.3390/foods14173124/s1.
