# Supplementary material for: Beverage Consumption in Reproductive-Age and Postmenopausal Mexican Women: Habits and Associated Factors
Source: Foods. 2025 Sep 6;14(17):3124. doi: 10.3390/foods14173124 (PMC12427969; doi:10.3390/foods14173124)
Supplement: Supplementary file 1 [file foods-14-03124-s001.zip › foods-3799563-supplementary.pdf]

Supplementary Table S1. Categorization of beverages according to the Guide for a Healthy Hydration of the Spanish Society of Community Nutrition [16].

Categories	Beverages
Category 1	Plain water
Category 2	Flavored beverages
Category 3	Fruit juices, diet soda, milk, yogurt, coffee and tea (usually with sugar)
Category 4	Regular soda, energy drinks
Category 5	Beer, wine, alcoholic distilled beverages
